# Supplementary material for: Effects of Brines and Containers on Flavor Production of Chinese Pickled Chili Pepper (Capsicum frutescens L.) during Natural Fermentation
Source: Foods. 2022 Dec 25;12(1):101. doi: 10.3390/foods12010101 (PMC9818826; doi:10.3390/foods12010101)
Supplement: Supplementary file 1 [file foods-12-00101-s001.zip › foods-2095931-supplementary.pdf]

Supplementary Table S1 Free amino acids content of pickled chili pepper processed by different procedures during 30 days' fermentation.

Content (mg/100g)	PFB						PAB					
	D0	D1	D4	D7	D14	D30	D0	D1	D4	D7	D14	D30
**Asp**	15.35±0.13^{d}	15.36±0.06^{d}	45.89±0.81^{a}	37.80±0.80^{bc}	38.39±0.56^{b}	36.37±0.13^{c}	13.19±0.41^{e}	18.91±0.40^{d}	61.33±1.21^{a}	47.59±0.64^{b}	45.93±1.16^{b}	40.87±0.07^{c}
**Thr**	112.76±2.39^{b}	123.53±0.73^{a}	94.37±0.53^{d}	80.15±1.69^{e}	103.34±1.50^{c}	50.79±0.08^{f}	117.35±4.29^{bc}	122.90±2.26^{b}	158.21±2.40^{a}	110.97±5.32^{c}	120.94±1.68^{b}	57.23±0.44^{d}
**Ser**	24.90±0.98^{cd}	23.73±0.26^{d}	31.25±0.78^{b}	25.21±0.56^{cd}	35.42±0.26^{a}	25.71±0.13^{c}	20.42±0.50^{e}	25.58±0.17^{d}	39.96±0.93^{a}	33.34±0.47^{b}	34.82±0.64^{b}	30.04±0.03^{c}
**Glu**	16.15±0.69^{d}	12.07±0.11^{e}	20.55±0.17^{c}	23.54±0.95^{b}	25.34±1.45^{ab}	26.23±0.59^{a}	15.04±0.52^{d}	14.15±0.13^{d}	27.84±0.48^{b}	30.02±0.78^{a}	25.71±0.48^{c}	26.95±0.65^{bc}
**Gly**	4.41±0.10^{f}	6.07±0.06^{e}	9.39±0.15^{d}	9.76±0.18^{c}	13.28±0.04^{a}	11.99±0.08^{b}	3.44±0.12^{c}	5.48±0.06^{b}	13.64±0.27^{a}	13.65±0.77^{a}	14.42±0.25^{a}	14.24±0.16^{a}
**Ala**	22.63±1.58^{e}	24.84±0.24^{d}	32.54±0.79^{b}	29.62±0.59^{c}	38.58±0.11^{a}	30.17±0.12^{c}	17.79±0.57^{e}	27.73±0.24^{d}	45.13±0.87^{a}	40.27±0.22^{b}	39.04±0.79^{b}	35.86±0.39^{c}
**Cys**	0.00±0.00^{e}	1.13±0.01^{d}	1.91±0.10^{c}	2.11±0.04^{b}	2.92±0.06^{a}	2.02±0.04^{bc}	0.49±0.04^{f}	0.82±0.02^{e}	2.59±0.06^{c}	3.43±0.05^{a}	3.04±0.04^{b}	2.24±0.05^{d}
**Val**	12.65±0.24^{e}	16.31±0.15^{d}	23.49±0.41^{c}	23.76±0.46^{c}	30.42±0.16^{a}	25.03±0.11^{b}	12.41±0.49^{e}	16.76±0.24^{d}	31.62±0.59^{b}	33.29±0.27^{a}	33.22±0.64^{a}	29.19±0.13^{c}
**Met**	1.51±0.06^{f}	3.84±0.03^{e}	8.37±0.16^{d}	9.71±0.18^{c}	12.05±0.23^{a}	10.89±0.11^{b}	1.30±0.03^{d}	3.41±0.09^{c}	11.84±0.25^{b}	12.59±0.20^{a}	11.83±0.06^{b}	12.02±0.19^{b}
**Ile**	4.05±0.11^{e}	6.42±0.07^{d}	13.54±0.21^{c}	13.64±0.26^{c}	17.45±0.10^{a}	15.54±0.09^{b}	4.04±0.14^{e}	6.63±0.06^{d}	17.22±0.34^{c}	19.20±0.12^{a}	18.76±0.34^{ab}	18.52±0.02^{b}
**Leu**	5.31±0.08^{f}	13.37±0.13^{e}	27.79±0.45^{d}	31.08±0.63^{c}	39.73±0.24^{a}	36.90±0.24^{b}	4.55±0.23^{d}	11.37±0.07^{c}	39.98±0.80^{b}	42.17±0.32^{a}	41.61±0.76^{a}	41.53±0.13^{a}

**Tyr**	5.80±0.16^{e}	11.84±0.13^{d}	20.56±0.27^{c}	20.99±0.54^{c}	25.44±0.18^{a}	23.70±0.27^{b}	6.76±0.33^{e}	11.96±0.18^{d}	28.39±0.57^{a}	29.14±0.19^{a}	27.05±0.39^{b}	25.98±0.13^{c}
**Phe**	6.41±0.12^{e}	18.96±0.14^{d}	32.88±0.47^{c}	34.77±0.63^{b}	38.09±1.28^{a}	35.34±0.23^{b}	6.93±0.30^{d}	16.62±0.23^{c}	44.84±0.95^{a}	44.14±0.30^{a}	40.39±1.43^{b}	39.09±0.60^{b}
**Lys**	9.57±0.12^{f}	19.23±0.14^{e}	31.21±0.47^{d}	33.05±0.65^{c}	38.91±0.25^{a}	36.53±0.23^{b}	9.84±0.39^{e}	16.87±0.16^{d}	42.56±0.85^{b}	45.03±0.24^{a}	42.51±0.79^{b}	40.44±0.07^{c}
**His**	6.29±0.78^{d}	8.95±0.06^{c}	12.52±0.05^{a}	11.58±0.24^{b}	12.77±0.09^{a}	11.31±0.12^{b}	7.80±0.22^{d}	10.86±0.09^{c}	16.24±0.35^{a}	15.88±0.24^{a}	15.90±0.23^{a}	12.41±0.07^{b}
**Arg**	8.28±0.30^{f}	13.41±0.10^{e}	23.83±0.45^{d}	26.58±0.57^{c}	31.72±0.28^{a}	30.07±0.20^{b}	8.42±0.26^{e}	16.08±0.04^{d}	32.55±0.76^{c}	36.71±0.41^{a}	37.54±0.61^{a}	34.33±0.10^{b}
**Pro**	6.25±0.23^{e}	9.77±0.10^{d}	17.69±0.31^{b}	17.25±0.18^{b}	22.08±0.69^{a}	16.15±0.29^{c}	4.96±0.18^{d}	9.81±0.11^{c}	20.85±0.22^{a}	21.39±0.51^{a}	21.51±0.52^{a}	17.89±0.29^{b}
**Umami FAA**	31.50±0.82^{c}	27.42±0.13^{d}	66.44±0.93^{a}	61.34±1.72^{b}	63.72±1.63^{ab}	62.59±0.72^{b}	28.22±0.90^{f}	33.07±0.53^{e}	89.17±1.69^{a}	77.61±0.88^{b}	71.64±0.73^{c}	67.82±0.69^{d}
**Bitter FAA**	58.36±1.83^{f}	108.49±0.38^{e}	185.83±0.25^{d}	195.46±3.94^{c}	234.54±0.68^{a}	214.42±1.42^{b}	60.75±2.26^{e}	107.15±0.90^{d}	253.40±5.20^{b}	265.56±1.96^{a}	256.97±2.35^{b}	241.50±0.35^{c}
**Sweet FAA**	166.20±5.08^{c}	182.01±1.28^{b}	175.92±0.79^{b}	154.45±3.15^{d}	202.67±2.03^{a}	129.55±0.22^{e}	160.31±5.37^{d}	185.11±2.23^{c}	268.77±3.66^{a}	210.81±5.24^{b}	221.05±2.83^{b}	149.37±1.09^{e}
**Total FAA**	262.30±7.66^{e}	328.82±0.99^{d}	447.79±1.60^{b}	430.61±8.95^{c}	525.93±3.93^{a}	424.74±2.60^{c}	254.72±7.42^{e}	335.96±3.58^{d}	634.78±10.33^{a}	578.81±3.14^{b}	574.21±5.91^{b}	478.82±1.26^{c}
	**JFB**						**JAB**					
	**D0**	**D1**	**D4**	**D7**	**D14**	**D30**	**D0**	**D1**	**D4**	**D7**	**D14**	**D30**
**Asp**	17.68±0.10^{c}	10.94±0.16^{e}	27.11±0.14^{a}	20.59±0.25^{b}	16.09±0.38^{d}	11.34±0.20^{e}	11.44±0.40^{c}	26.72±0.32^{b}	34.86±1.94^{a}	34.04±0.77^{a}	24.54±1.97^{b}	24.95±0.36^{b}
**Thr**	53.15±1.54^{c}	66.69±0.51^{b}	71.51±0.38^{a}	42.16±0.45^{d}	26.32±0.49^{e}	11.56±0.40^{f}	67.86±0.63^{b}	72.38±0.80^{b}	99.06±10.37^{a}	68.47±1.93^{b}	24.91±1.32^{c}	12.21±0.26^{d}

**Ser**	11.90±0.13^{d}	16.53±0.07^{b}	20.87±0.06^{a}	13.18±0.10^{c}	11.47±0.35^{d}	6.74±0.17^{e}	16.20±0.34^{d}	19.77±0.30^{c}	22.42±1.77^{b}	25.91±0.69^{a}	17.54±1.30^{cd}	16.62±0.11^{d}
**Glu**	11.33±0.27^{c}	10.79±0.05^{c}	19.89±0.11^{a}	14.74±0.27^{b}	11.26±0.46^{c}	7.66±0.06^{d}	19.49±0.52^{b}	14.89±0.50^{cd}	20.59±0.72^{b}	22.76±0.75^{a}	16.49±0.90^{c}	13.16±0.29^{d}
**Gly**	2.32±0.00^{d}	4.23±0.05^{c}	6.33±0.03^{a}	4.52±0.02^{b}	2.27±0.11^{d}	1.00±0.06^{e}	2.20±0.05^{c}	4.25±0.12^{bc}	7.88±0.26^{a}	7.32±0.24^{a}	6.45±2.10^{ab}	7.22±0.15^{a}
**Ala**	14.85±0.15^{e}	20.19±0.28^{b}	27.18±0.13^{a}	18.90±0.12^{c}	16.08±0.33^{d}	9.15±0.16^{f}	18.89±0.47^{e}	21.80±0.62^{d}	28.47±1.21^{b}	33.28±0.76^{a}	24.49±0.38^{c}	20.19±0.60^{de}
**Cys**	0.00±0.00^{f}	0.77±0.04^{d}	1.25±0.04^{a}	0.96±0.03^{c}	1.04±0.01^{b}	0.68±0.02^{e}	0.00±0.00^{d}	0.84±0.07^{c}	1.29±0.07^{ab}	1.45±0.05^{a}	1.05±0.03^{bc}	1.02±0.18^{c}
**Val**	8.14±0.07^{d}	11.45±0.05^{c}	16.76±0.09^{a}	12.81±0.11^{b}	12.80±0.15^{b}	8.13±0.09^{d}	7.72±0.16^{f}	12.77±0.25^{e}	20.45±0.81^{b}	24.96±0.57^{a}	18.40±0.47^{c}	16.56±0.18^{d}
**Met**	0.63±0.01^{f}	2.05±0.04^{e}	5.93±0.11^{a}	4.73±0.02^{c}	4.93±0.11^{b}	3.06±0.03^{d}	0.75±0.02^{e}	3.34±0.06^{d}	7.53±0.35^{b}	9.85±0.11^{a}	7.64±0.18^{b}	6.01±0.09^{c}
**Ile**	3.45±0.02^{f}	4.65±0.04^{d}	8.53±0.08^{a}	6.56±0.08^{c}	7.38±0.08^{b}	4.19±0.05^{e}	2.54±0.05^{e}	5.48±0.13^{d}	10.34±0.52^{bc}	14.90±0.43^{a}	10.88±0.28^{b}	9.52±0.14^{c}
**Leu**	3.00±0.05^{f}	7.41±0.12^{e}	20.67±0.14^{a}	16.01±0.20^{c}	18.05±0.24^{b}	10.55±0.14^{d}	2.40±0.06^{e}	11.40±0.19^{d}	24.25±1.14^{b}	34.65±1.00^{a}	25.87±0.69^{b}	21.62±0.36^{c}
**Tyr**	5.52±0.08^{e}	7.91±0.10^{d}	15.65±0.11^{a}	13.06±0.20^{b}	12.72±0.28^{b}	8.49±0.04^{c}	4.28±0.08^{e}	10.44±0.08^{d}	16.91±0.71^{b}	22.04±0.63^{a}	16.69±0.47^{b}	12.68±0.19^{c}
**Phe**	5.08±0.08^{e}	9.45±0.04^{d}	27.51±0.18^{a}	22.72±0.31^{b}	23.88±1.43^{b}	14.97±0.13^{c}	3.59±0.09^{d}	22.68±4.27^{c}	30.82±0.53^{b}	41.27±1.06^{a}	31.79±0.65^{b}	24.25±0.49^{c}
**Lys**	8.71±0.10^{f}	13.43±0.13^{d}	24.16±0.16^{a}	18.53±0.17^{c}	19.08±0.20^{b}	12.10±0.11^{e}	6.01±0.18^{e}	15.23±0.22^{d}	26.50±1.25^{b}	31.21±0.58^{a}	24.87±0.63^{b}	20.59±0.26^{c}
**His**	4.18±0.48^{c}	6.21±0.04^{b}	8.29±0.08^{a}	5.94±0.11^{b}	4.24±0.11^{c}	2.59±0.07^{d}	4.27±0.08^{d}	6.97±0.03^{b}	6.99±0.37^{b}	10.87±0.29^{a}	5.50±0.14^{c}	5.27±0.90^{cd}

**Arg**	4.81±0.08^{f}	8.18±0.06^{d}	16.64±0.12^{a}	12.25±0.21^{c}	12.83±0.37^{b}	7.05±0.18^{e}	4.17±0.14^{e}	11.73±0.19^{d}	19.20±0.78^{b}	26.34±0.88^{a}	18.82±0.58^{b}	15.55±0.23^{c}
**Pro**	4.02±0.27^{d}	6.58±0.17^{c}	12.31±0.11^{a}	9.33±0.15^{b}	12.65±1.18^{a}	9.51±0.28^{b}	4.69±0.36^{d}	9.23±0.41^{c}	13.96±0.33^{b}	17.46±0.27^{a}	13.18±2.74^{b}	15.48±0.23^{ab}
**Umami FAA**	29.01±0.35^{c}	21.74±0.13^{e}	47.00±0.22^{a}	35.33±0.51^{b}	27.35±0.83^{d}	19.00±0.24^{f}	30.93±0.50^{c}	41.61±0.75^{b}	55.44±1.31^{a}	56.80±1.39^{a}	41.03±2.86^{b}	38.11±0.25^{b}
**Bitter FAA**	42.88±0.57^{d}	68.70±0.18^{c}	138.20±0.77^{a}	107.88±1.36^{b}	110.98±2.74^{b}	68.06±0.75^{c}	34.98±0.51^{e}	96.69±3.28^{d}	155.47±4.57^{b}	206.24±5.23^{a}	152.81±3.90^{b}	126.04±1.85^{c}
**Sweet FAA**	82.85±1.32^{c}	109.70±0.37^{b}	131.83±0.52^{a}	83.49±0.65^{c}	61.07±0.50^{d}	31.51±0.71^{e}	105.90±1.44^{d}	121.53±1.86^{c}	165.35±10.61^{a}	144.81±3.50^{b}	81.02±5.22^{e}	62.25±0.42^{f}
**Total FAA**	158.76±1.42^{e}	207.49±0.40^{d}	330.60±1.38^{a}	236.99±2.42^{b}	213.08±2.21^{c}	128.77±1.72^{f}	176.49±1.85^{f}	269.89±1.11^{d}	391.50±8.08^{b}	426.76±10.36^{a}	289.10±10.43^{c}	242.90±2.11^{e}

Different superscript letters in the same row of PFB or PAB and JFB or JAB indicate significantly difference (n = 3, $P < 0.05$).
